# Development of RLK-Unet: a clinically favorable deep learning algorithm for brain metastasis detection and treatment response assessment

**DOI:** 10.3389/fonc.2023.1273013

**Published:** 2024-01-15

**Authors:** Seungyeon Son, Bio Joo, Mina Park, Sang Hyun Suh, Hee Sang Oh, Jun Won Kim, Seoyoung Lee, Sung Jun Ahn, Jong-Min Lee

**Affiliations:** ^1^ Department of Artificial Intelligence, Hanyang University, Seoul, Republic of Korea; ^2^ Department of Radiology, Gangnam Severance Hospital, Yonsei University, College of Medicine, Seoul, Republic of Korea; ^3^ Department of Radiation Oncology, Gangnam Severance Hospital, Yonsei University, College of Medicine, Seoul, Republic of Korea; ^4^ Division of Medical Oncology, Department of Internal Medicine, Gangnam Severance Hospital, College of Medicine, Yonsei University, Seoul, Republic of Korea; ^5^ Department of Biomedical Engineering, Hanyang University, Seoul, Republic of Korea

**Keywords:** deep learning algorithm, brain metastasis, detection, segmentation, treatment response

## Abstract

**Purpose/objective(s):**

Previous deep learning (DL) algorithms for brain metastasis (BM) detection and segmentation have not been commonly used in clinics because they produce false-positive findings, require multiple sequences, and do not reflect physiological properties such as necrosis. The aim of this study was to develop a more clinically favorable DL algorithm (RLK-Unet) using a single sequence reflecting necrosis and apply it to automated treatment response assessment.

**Methods and materials:**

A total of 128 patients with 1339 BMs, who underwent BM magnetic resonance imaging using the contrast-enhanced 3D T1 weighted (T1WI) turbo spin-echo black blood sequence, were included in the development of the DL algorithm. Fifty-eight patients with 629 BMs were assessed for treatment response. The detection sensitivity, precision, Dice similarity coefficient (DSC), and agreement of treatment response assessments between neuroradiologists and RLK-Unet were assessed.

**Results:**

RLK-Unet demonstrated a sensitivity of 86.9% and a precision of 79.6% for BMs and had a DSC of 0.663. Segmentation performance was better in the subgroup with larger BMs (DSC, 0.843). The agreement in the response assessment for BMs between the radiologists and RLK-Unet was excellent (intraclass correlation, 0.84).

**Conclusion:**

RLK-Unet yielded accurate detection and segmentation of BM and could assist clinicians in treatment response assessment.

## 1 Introduction

Lung cancer is the most frequent source of brain metastases (BMs), and 30%–50% of patients with lung cancer develop BMs during the course of the disease (1). As a result, brain magnetic resonance imaging (MRI) has become an important part of staging and treatment planning for lung cancer. Many guidelines recommend brain MRI for the screening and follow-up of BMs in advanced non-small lung cancer or small cell lung cancer (2, 3). However, the detection of small BMs and an accurate assessment of treatment response require tedious effort by radiologists. In addition, stereotactic radiosurgery has become popular in the treatment of BMs; therefore, manual segmentation of BMs has significantly increased the workload of radiosurgeons (4, 5).

In this context, recent studies (6–8) have implemented deep learning models, particularly deep convolutional neural networks (CNNs), for the automatic detection and segmentation of BMs, and have reported promising results with sensitivities of up to 90% and Dice coefficients of up to 0.8. However, the studies often report a substantial number of false-positive (FP) results and low sensitivity in detecting small BMs. Moreover, their segmentation methods were based on multiparametric scans such as the T1-weighted image T2-weighted image (T2WI), contrast-enhanced T1WI, and fluid-attenuated inversion recovery (FLAIR). However, these methods are not always favorable because additional sequences may increase the scan time and are often acquired with a larger thickness and lower resolution, which may add uncertainty to the segmentation. A few studies (9, 10) have used a single modality—in particular, the contrast-enhanced 3D gradient echo (GRE) T1WI sequence. However, recent studies (11, 12) have demonstrated that the three-dimensional (3D) black blood (BB) T1WI sequence is superior to the 3D GRE T1WI sequence in detecting small BMs by suppressing intraluminal blood signals. In a subsequent study, deep learning (DL)-based methods for BM detection and segmentation, utilizing the 3D BB T1WI sequence, demonstrated a better performance advantage over methods employing the 3D GRE T1WI sequence (8). In that study, the sensitivity for detecting brain metastases (BM) on 3D BB T1WI was higher at 92.6% compared to the sensitivity on 3D GRE T1WI, which stood at 76.8%.

Another limitation of previous studies is that internal necrosis was included in the BM segmentation. BM necrosis may represent a by-product of chemotherapy or radiation therapy (13, 14). The Response Assessment in Neuro-Oncology Brain Metastases (RANO-BM) criteria also recommend that these necrotic or cystic cavities should not be measured for determining a response (15). Thus, previous BM segmentation algorithms that included solid components and necrosis may lead to inappropriate treatment assessment.

The aims of our study were two-fold (1): to assess whether a DL algorithm using a single modality, 3D BB T1WI, has promising performance for the detection and segmentation of BMs and (2) to investigate whether the volumetric assessment using our developed DL algorithm, excluding necrosis, is comparable to the conventional assessment based on the RANO-BM criteria.

## 2 Materials and methods

### 2.1 Participants

This retrospective study was approved by our institutional review board, which waived the requirement for informed consent. We retrospectively searched the electronic medical records to identify patients with lung cancer who underwent brain MRI to evaluate BMs diagnosed between April 2017 and December 2021. For the segmentation of BMs between April 2017 and October 2020, 128 consecutive patients with newly developed 1339 BMs were included (Dataset 1). For the assessment of the treatment response between November 2020 and December 2021, 59 consecutive patients with 629 BMs were included (Dataset 2). The detailed inclusion and exclusion criteria are described in **Supplementary Material S1**. Histopathological diagnoses of lung cancer were determined by using bronchoscopic, percutaneous needle-guided, or surgical biopsies in all patients.

### 2.2 MRI protocol

Routine MRIs for the evaluation of the BMs were acquired using the Siemens 3T Vida scanner (Siemens Healthineers, Erlangen, Germany) or the GE 3T Discovery MR750 scanner (GE Healthcare, Milwaukee, WI, USA). Our BM MRI protocol consisted of T1-weighted image (T1WI), T2-weighted image (T2WI), FLAIR, contrast-enhanced T1WI, and BB T1WI. Contrast-enhanced images were acquired after administering gadobutrol (0.2 mmol/kg; Gadovist, Bayer Schering Pharma; Berlin, Germany). Detailed MR parameters are provided in **Supplementary Material S2**.

### 2.3 BM segmentation

**Figure 1:**
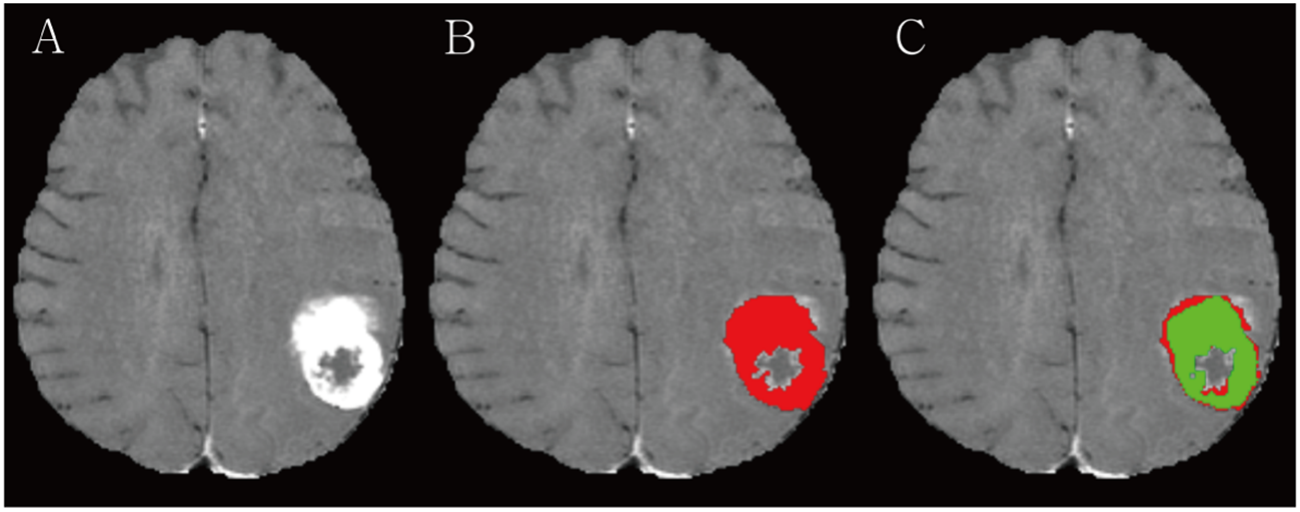
**(A)** A large necrotic brain metastasis (BM) is noted in left parietal lobe on contrast-enhanced 3D turbo spin-echo (TSE) black blood (BB) sequence. **(B)** Radiologist manually segmented BM avoiding a necrosis (red). **(C)** RLK-Unet algorithm predicted segmentation of BMs. Dice similarity coefficient (DSC) was 0.894.

The ground truths (GTs) in all BMs were carefully drawn by a radiologist with 8 years of clinical experience, while avoiding cystic or necrotic areas on contrast-enhanced BB T1WIs and referring to T1WIs, T2WIs, and contrast-enhanced T1WIs, by using the open-source software ITK-Snap, version 3.8.0 (available at www.itksnap.org) (**Figure 1**) (16). Another neuroradiologist with 14 years of clinical experience confirmed the segmented BMs or modified ambiguous cases.

### 2.4 Treatment response

The treatment response, based on the RANO-BM criteria, was independently assessed and classified into three categories by two radiologists (HSO and SJA, who had 4 years and 14 years of clinical experience, respectively) (15): complete response (CR), partial response/stable disease (PR/SD), and progressive disease (PD). Inconsistent cases were determined by a consensus between the two radiologists. The treatment response of the DL algorithm was based on the volumetric response by using the modified RANO-BM criteria (17). While the RANO-BM guidelines emphasize the significance of volumetric analysis, they do not provide specific criteria. Therefore, we took inspiration from the fundamental principles of the RANO-BM guidelines and defined volumetric criteria based on the established unidimensional recommendations, using spherical geometry. In this context, PD was defined as a volume increase of ≥ 72.8% in the present study compared to the baseline. This corresponds to a ≥ 20% increase in the diameter of a perfect sphere, aligning with the unidimensional RANO-BM criteria for progression.

### 2.5 Deep learning algorithm

**Figure 2:**
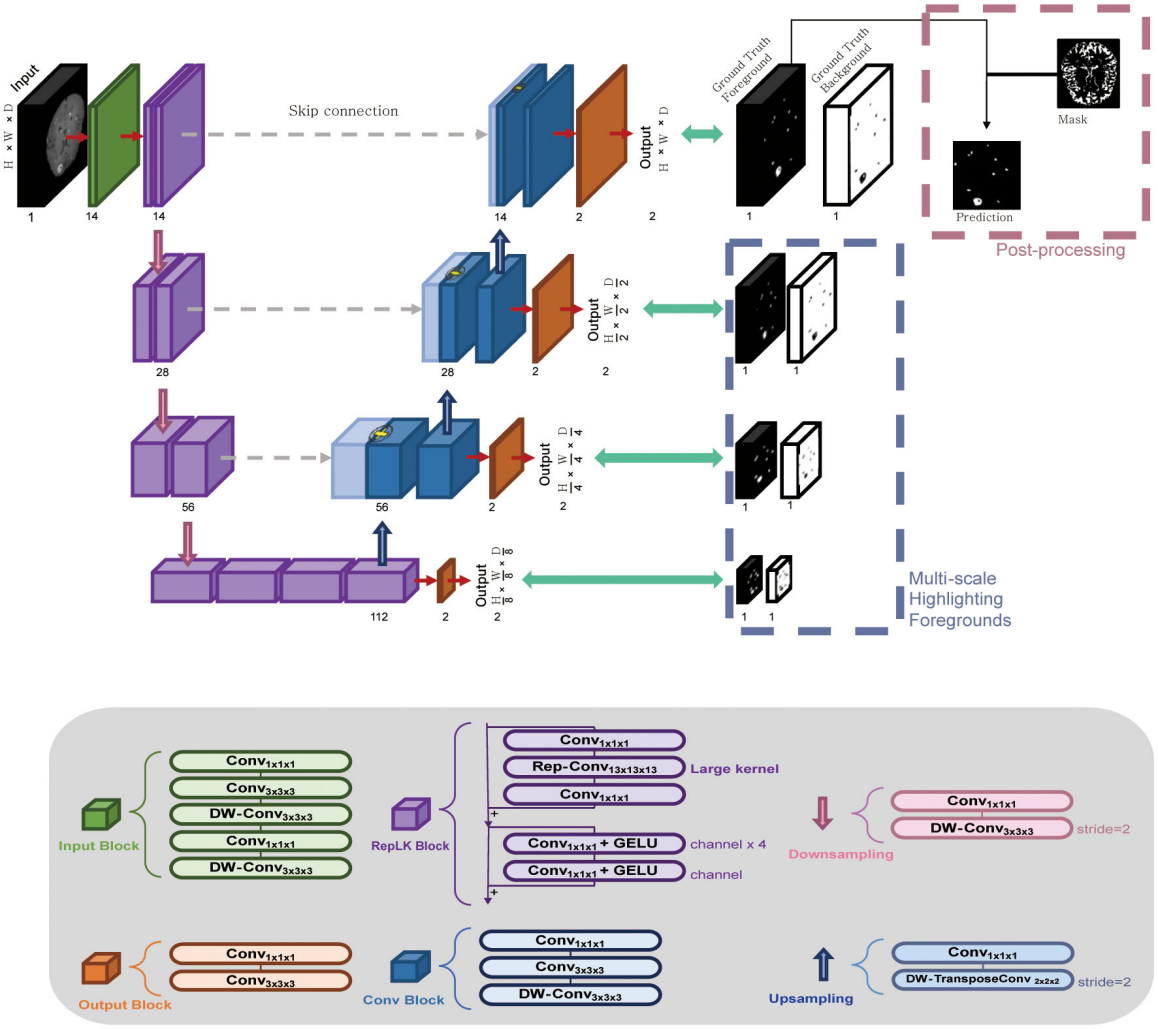
The workflow of the RLK-Unet algorithm, which includes a reparameterized large kernel and multiscale highlighting foregrounds. RLK-Unet has four layers in the encoder and the decoder, respectively. To capture information from a large region, a large kernel (13×13×13) was applied in the encoder and multiscale highlighting foregrounds were introduced in the decoder to improve the detection of brain metastases. Conv, convolution; DW-Conv, depth-wise convolution; GELU, Gaussian error linear unit.

The U-Net architecture is a powerful and flexible tool for image segmentation tasks, and its success has led to the development of many variations and extensions of the original architecture (18–20). In the current study, we propose a modified DL-based 3D U-Net architecture, named RLK-Unet, which incorporates re-parameterizing and multiscale highlighting foregrounds (MHFs), along with postprocessing (**Figure 2**). The training data for RKL-Unet consisted of contrast-enhanced 3D BB T1WIs as the input and the GT as the reference mask. The experiments were conducted by splitting Dataset 1 into five folds. In each round of the five-fold cross-validation procedure, four data folds were employed as the training cases, and the remaining fold was used for testing. Ten percent of the training samples were randomly selected for validation. Particularly, the stratified K-fold method was used to ensure an even distribution of small and large BMs in both the training and test sets (21). Details of the network configuration are provided in **Supplementary Material S3**.

RKL-Unet comprises an encoder that extracts the low-level features of the input data and a decoder that reconstructs the corresponding label map. Feature maps of the encoder are passed to the decoder by using skip connection, which concatenates the feature maps from the corresponding encoder layer to preserve the spatial information lost in the encoding stage (22). To improve the segmentation performance, we applied the guidelines proposed by Ding et al. (23) to the encoder of our architecture, which allowed us to build a large receptive field by using a large kernel. Thus, we used a large kernel structure (13 × 13 × 13) instead of a small kernel (3 × 3 × 3) that is typically used in U-Net models to extract feature maps through large receptive fields. Furthermore, we introduced MHFs into the U-Net architecture, highlighting foreground regions at multiple scales, which allowed the network to better differentiate between BMs and healthy brain tissue, even in situations in which lesions are small or have a low contrast (24). Additional postprocessing was conducted to eliminate blood vessels that were incompletely suppressed in the BB images and choroid-plexuses, which may mimic BMs and increase the number of FPs. We implemented the surface and choroid plexus mask to effectively reduce the number of FPs in the output. This process is conducted solely on the foreground channels of the output features of the trained model. Detailed postprocessing is described in **Supplementary Material S4**.

### 2.6 Statistical methods

Lesion-based sensitivity and precision were calculated to assess the detection performance of the DL algorithm. Sensitivity and precision were defined by using true-positive (TP), false-negative (FN), and FP metrics, as follows:


$$Sensitivity=\frac{TP}{TP+FN}$$

$$Precision=\frac{TP}{TP+FP}$$

Sensitivities were also evaluated with respect to the size of BMs (i.e., ≤10 mm or >10 mm). The automatic segmentation results were compared with the GT, using the Dice similarity coefficient (DSC) to investigate the segmentation performance of the DL algorithm. The DSC computes the overlap of the GT segmentation (V_g_) and automatic segmentation (V_s_), as follows:


$$DSC=\frac{2*\left|Vs\cap Vg\right|}{\left|Vs\right|\;+\;\left|Vg\right|}$$

Pearson’s correlation and Bland–Altman analysis were conducted to compare volumetric measurements of the GT and automatic segmentations (25, 26). Agreement between the neuroradiologist and the DL algorithm for treatment response was assessed by using the intra-class correlation coefficient (ICC) with a two-way random model of absolute agreement (27).

## 3 Results

### 3.1 Patient characteristics

In our study, a total of 186 patients diagnosed with lung cancer and brain metastases (BMs) were enrolled and categorized into two distinct groups. Dataset 1, designated for BM segmentation, comprised 128 patients with a mean age of 67.1 ± 9.9 years, consisting of 87 men and 41 women. Dataset 2, intended for response assessment, involved 58 pairs of sequential brain MRIs corresponding to 58 patients, with a mean age of 63.2 ± 9.5 years, including 35 men and 23 women. The average time interval between the baseline and follow-up MRI scans was 3.53 ± 1.32 months. Among the 58 lung cancer patients, the breakdown of treatments was as follows: 57% underwent whole-brain radiotherapy (WBRT), 19% received stereotactic radiosurgery (SRS) alone, 15% were on tyrosine kinase inhibitors alone, and 9% underwent a combination of SRS and WBRT. Comprehensive patient characteristics are detailed in **Table 1**. Distribution of small and large BMs in training and test sets during 5-fold cross-validation is summarized in the **Table 2**. Bar graph describes distribution of size of BMs across all folds (**Supplementary Materials S4**, **S5**).

**Table 1:** Patients’ characteristics.

Variable	Dataset for BM segmentation (n=128)	Dataset for response assessment (n=58)	Total (n=186)
Age (y, mean ± SD)	67.15 ± 9.86	63.21 ± 9.46	65.92 ± 9.88
Sex			
Female	41 (32.0%)	23 (39.7%)	64 (34.4%)
Male	87 (68.0%)	35 (60.3%)	122 (65.6%)
Number of BMs			
1	29 (22.7%)	10 (17.2%)	39 (21.0%)
2–5	47 (36.7%)	24 (41.4%)	71 (38.2%)
6–10	21 (16.4%)	11 (19.0%)	32 (17.2%)
>10	31 (24.2%)	13 (22.4%)	44 (23.7%)
Volume of BM, mm^3^ (mean ± SD)	694.13 ± 2057.07	419.01 ± 1192.27	608.34 ± 1832.81

BM, brain metastasis; SD, standard deviation.

Data are presented as the mean ± standard deviation or as numbers of patients (%).

**Table 2:** Distribution of small and large BMs in training and test sets during 5-fold cross validation.

	Training		Test	
	Small BM	Large BM	Small BM	Large BM
Number of BMs at fold 1	994 (84.1%)	187 (15.9%)	120 (75.9%)	38 (24.1%)
Number of BMs at fold 2	881 (81.7%)	197 (18.3%)	233 (89.2%)	28 (10.8%)
Number of BMs at fold 3	665 (82.5%)	141 (17.5%)	449 (84.2%)	84 (15.8%)
Number of BMs at fold 4	999 (84.3%)	186 (15.7%)	115 (74.6%)	39 (25.4%)
Number of BMs at fold 5	917 (82.9%)	189 (17.1%)	197 (84.5%)	36 (15.5%)
Mean	891 (83.1%)	180 (16.9%)	222 (83.1%)	45 (16.9%)

Small BM: Brain metastasis (≤10 mm in diameter).

Large BM: Brain metastasis (>10 mm in diameter).

Data are presented as numbers of patients (%).

### 3.2 Detection and segmentation performance of DL algorithms

The detection sensitivities and precisions of RLK-Unet are summarized in **Table 3**. RLK-Unet demonstrated a sensitivity of 86.9% and a precision of 79.6% for all BMs. False positive (FP) per scan was 1.76. In particular, we evaluated the predicted result from RLK-Unet, focusing on the assessment of segmentation performance that excludes necrosis and the detection of small BMs. The predicted results of RLK-Unet were analyzed by categorizing the BMs into two groups using a diameter threshold of 10 mm. The sensitivity and precision for the detection of small BMs (≤10 mm) were 80.84% and 87.39% respectively, whereas the sensitivity and precision for large BMs (>10 mm) was 98.66% and 91.10% respectively. In addition, FP per scan for small BMs was relatively higher (1.6) than that for large BM (0.15).

**Table 3:** Detection and segmentation performance of RLK-Unet.

	Small BM	Large BM	All
Detection			
Sensitivity (%)	80.84 ± 7.32	98.66 ± 1.26	86.90 ± 4.07
Missed BM/patient (%)	1.67 ± 0.93	0.03 ± 0.05	1.71 ± 0.86
FP/scan	1.60 ± 0.19	0.15 ± 0.10	1.76 ± 0.22
Precision (%)	78.39 ± 8.27	91.10 ± 6.18	79.60 ± 6.46
Segmentation			
DSC	0.54 ± 0.08	0.85 ± 0.03	0.66 ± 0.02

BM, brain metastasis; DSC, Dice similarity coefficient; FP, false-positive.

**Figure 3:**
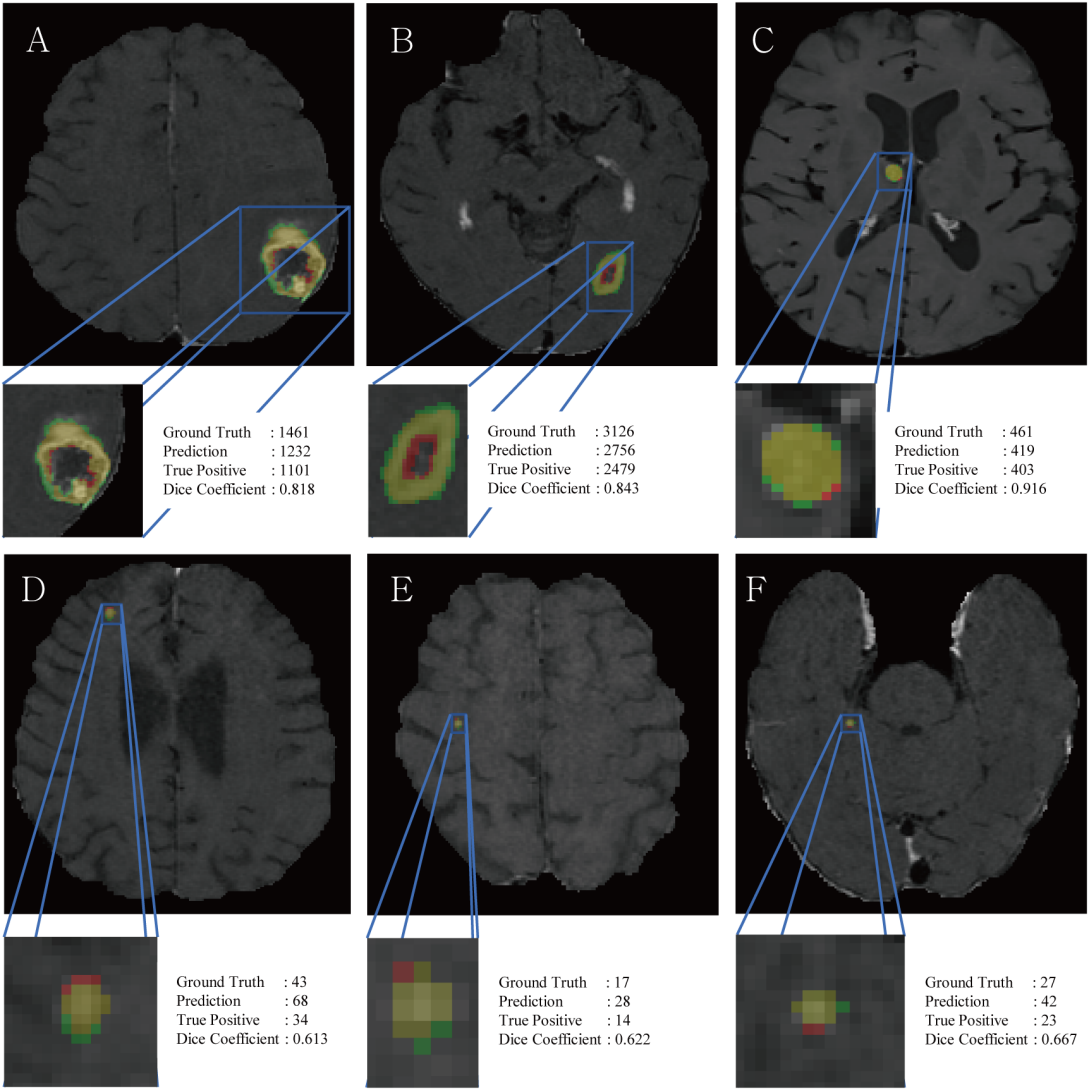
Representative figures, voxel counts of ground truth, prediction, true positives and DSC scores of large BM **(A–C)** and small BM **(D–F)**. Red and green colors indicate false positives and false negatives, respectively, while the yellow color represents true positives.

**Figure 4:**
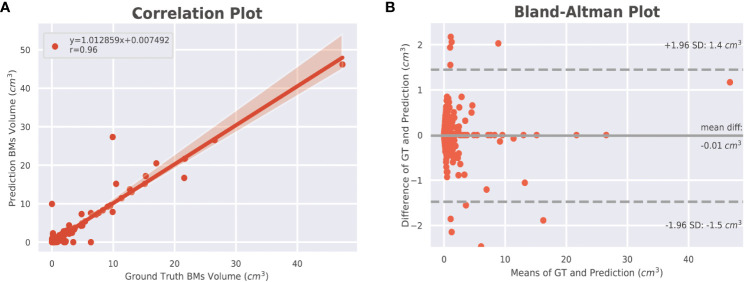
Volumetric correlations between ground truth and automated segmentations of BMs. **(A)** Pearson correlation (*r*) at the lesion level. The shaded area indicates the 95% confidence interval (95% CI) of the fitted line (*y* = the regression equation). **(B)** Bland–Altman analysis at the lesion level. The solid line indicates the mean difference between the two segmentations, whereas the dotted lines indicate the 95% limit of agreement. BM, brain metastasis; GT, ground truth.

The DSC for all BMs was 0.663, whereas the DSCs for the large and small BMs were 0.851 and 0.535, respectively (**Figure 3**). **Figure 4** displays the volumetric correlation between the GT and the automated segmentation. The Pearson’s correlation coefficient (*r*) was 0.96, which indicated a strong positive correlation between the two sets. Bland–Altman analysis findings also demonstrated excellent agreement with a difference of 0.01 cm^3^ between the two sets of results. These results confirmed the accuracy and reliability of the proposed algorithm.

The detection and segmentation performance of each step of RLK-Unet are presented in **Supplementary Material S7**. The use of encoder blocks with a large kernel (13 × 13 × 13) in our 3D U-Net architecture improved the sensitivity for detecting BMs to 88.3%, compared to the sensitivity of 84.5% that was achieved using smaller kernel sizes. The application of MHFs increased the precision from 68.4% to 73.9%. After postprocessing, the precision further improved from 73.9% to 79.6%.

### 3.3 Agreements in the response assessment for BMs

**Figure 5:**
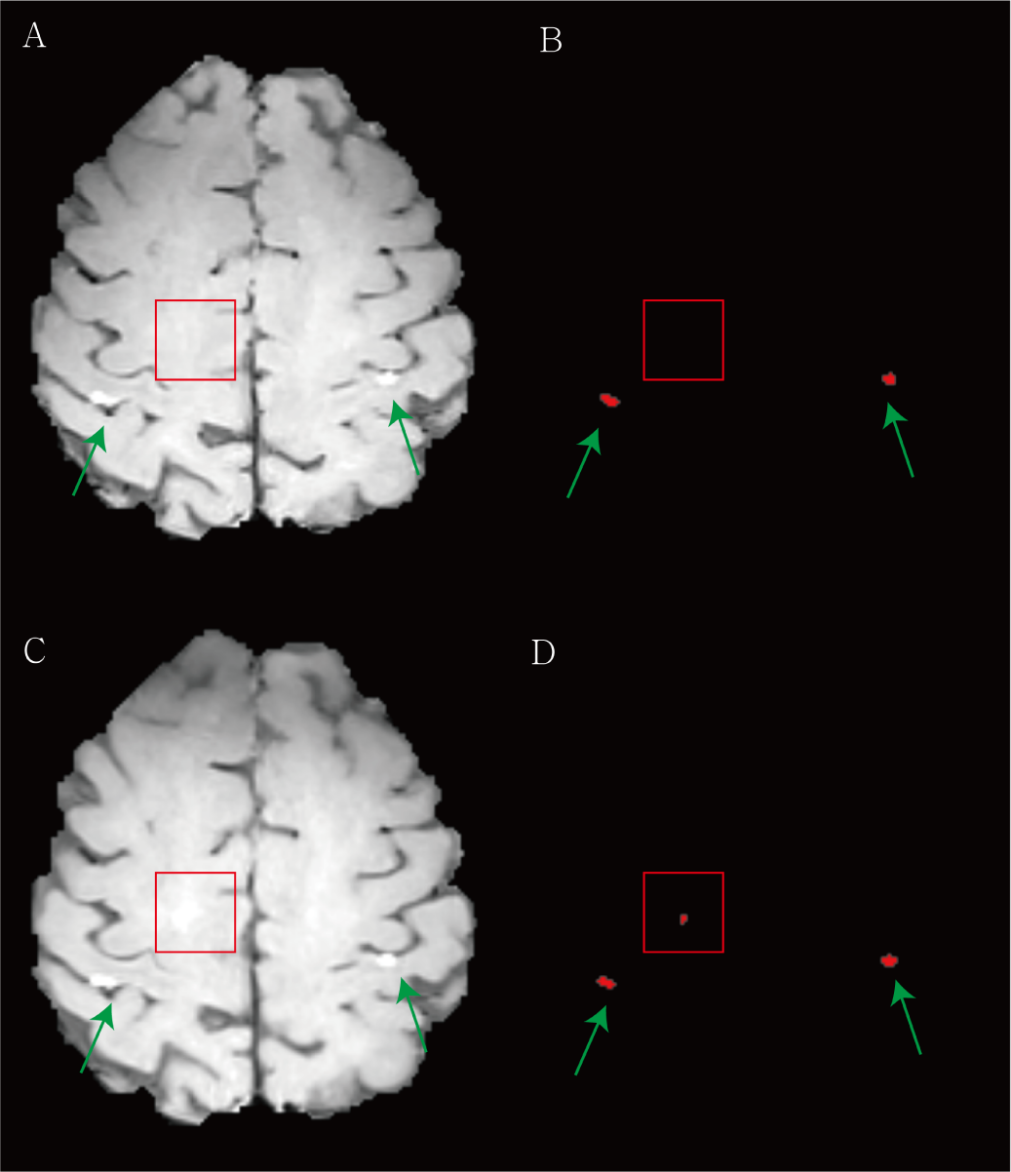
Examples of the overestimation of treatment response for brain metastasis by the deep learning (DL) algorithm. **(A)** The baseline contrast-enhanced three-dimensional (3D) turbo spin-echo (TSE) black blood (BB) T1-weighted image (T1WI) shows two metastases in both parietal cortices (green arrows). **(B)** Our DL algorithm predicted two corresponding metastases. **(C)** In the follow-up 3D TSE BB T1WI, the radiologist classified this case as stable. **(D)** The DL algorithm regarded the equivocal enhancement (red box) in right deep white matter as a new lesion and assessed this finding as progression.

**Figure 6:**
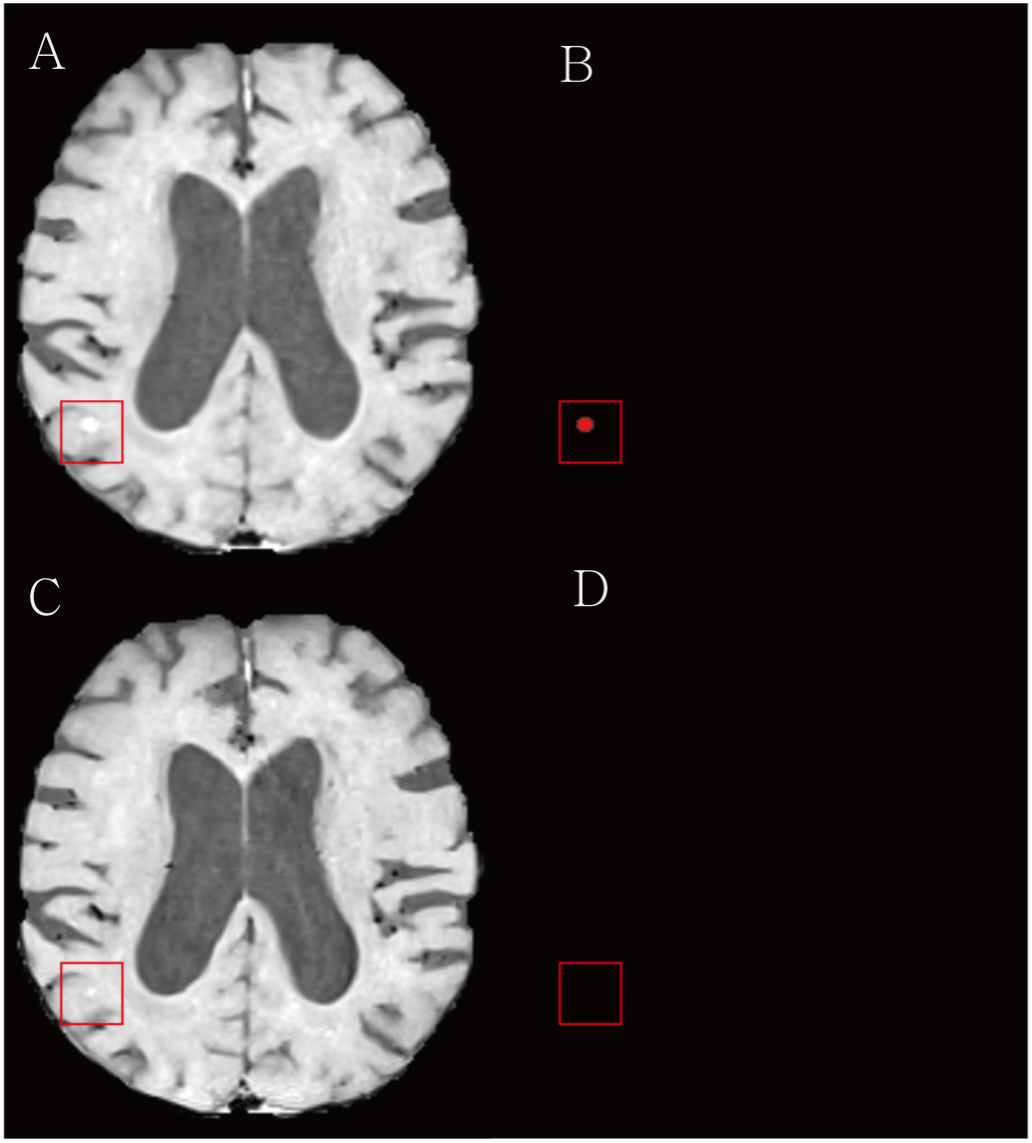
Examples of the underestimation of treatment response for brain metastasis by the deep learning (DL) algorithm. **(A)** The baseline contrast-enhanced three-dimensional (3D) turbo spin-echo (TSE) black blood (BB) T1WI shows a metastasis in the right parieto-temporal lobe (red box). **(B)** Our DL algorithm predicted a corresponding metastasis. **(C)** On the follow up 3D TSE BB T1WI, the radiologist classified this case as a partial response/stable. **(D)** The DL algorithm missed a remaining tumor and assessed this case as complete remission.

The agreement in the response assessment of BMs between the radiologists and the DL algorithm was excellent [ICC = 0.84; 95% confidence interval (CI), 0.75-0.91]. Response assessment for BM in 87.9% (51/58) of patients was agreed on by the radiologist and the DL algorithm (**Table 4**). The DL algorithm overestimated the response assessment in 6.8% (4/58) of patients (**Figure 5**) in which all PR/SD cases were misclassified as PD, and underestimated the response assessment in 5.1% (3/58) of patients (**Figure 6**), in which one PD case was misinterpreted as PR/SD and two PR/SD cases were misinterpreted as CR.

**Table 4:** Response assessment by the radiologists and by the deep learning algorithm.

		Response assessment by the deep learning algorithm		
		CR	PR/SD	PD
Response assessment by radiologist	CR	3	0	0
	PR/SD	1	24	1
	PD	0	1	28

CR, complete response; PR/SD, partial response/stable disease; PD, progressive disease.

## 4 Discussion

RLK-Unet for the detection and segmentation of BMs has two clinically favored features that previous models have rarely tried. First, RLK-Unet was based only on a single modality, 3D BB T1WI. Second, RLK-Unet segments the solid part of the tumor to avoid necrosis. Nevertheless, RLK-Unet exhibited promising performance for detection and segmentation. Moreover, the volumetric assessment by RLK-Unet strongly agreed with that of the response assessment by the radiologist, based on the RANO-BM criteria. Thus, our model is expected to facilitate clinical workflow and to potentially improve patient outcomes via a volumetric assessment of the treatment response.

While earlier studies demonstrated high sensitivity in the detection of BMs, surpassing 80%, they were accompanied by a significant number of FPs and, consequently, exhibited low precision, as indicated in **Table 5** (7, 10, 28, 29). Subsequent research, incorporating multiple modalities, showed improved performance with sensitivities ranging from 82% to 100% and reducing the FP rate to between 0.6 and 1.5 per scan (6, 8, 30). Notably, a recent study, utilizing a single modality, introduced a novel loss function and integrated temporal prior information, achieving exceptional results (sensitivity: 84%; precision: 99%; FP rate: 1) (31). Another extensive study also reported remarkable outcomes (sensitivity: 88.4%; precision: 90.1%; FP rate: 0.4) (32). However, it is essential to acknowledge that most of these studies did not address the critical issue of excluding internal necrosis, which is pivotal for accurate volumetric assessment of tumor burden. In contrast, our RLK-Unet successfully addressed the exclusion of necrotic regions within BMs, achieving outstanding performance (sensitivity: 86.9%; precision: 79.6%; FP rate: 1.8). To achieve this, we implemented several strategies within our DL algorithm, enabling us to maintain high sensitivity while concurrently reducing the FP rate.

**Table 5:** Comparison of published DL-based BMs detection and segmentation performance.

Author	Number of patients (train/test)	Number of BMs (train/test)	BM size		Acquisition image	DL model	Performance				Excluding necrosis
			Diameter (mm)	Volume (cm^3^)			Sensitivity (%)	FP/scan	Precision (%)	DSC	
Charron et al., 2018 (28)	164/18	374/38	8.1	2.4	Multisequence	DeepMedic	94	12.7/patient		0.77	Y
Grøvik et al., 2020 (7)	105/51	-/856	2-40	–	Multisequence	GoogLe Net	50:<7 mm 80:<22 mm 100: ≥ 22 mm	8 ± 13	79 ± 20	0.79 ± 0.12	N
Xue et al., 2020 (10)	1201	–	2-45	0.1-23.8	T1 CE (T1 GRE)	BMDS Net	96 (≥6 mm)	–	*-*	0.85 ± 0.08 (≥6 mm)	N
Zhou et al., 2020 (29)	212/54	–	10 ± 8	–	T1 CE (T1 GRE)	Single-shot Detector	15:<3mm 70: 3~6mm 98: ≥ 6 mm	3-4 (≥ 6 mm)	100:<3mm 35: 3~6mm 36≥ 6 mm	–	N
Jünger et al., 2021 (6)	66/17	248/67	–	1.0 ± 4.2	Multisequence	DeepMedic	86 ± 4	1.5	-68.7	0.75 ± 0.02	N
Park et al., 2021 (8)	188/94	917/203	9.9 ± 10.9	1.6 ± 6.5	Multisequence	U-Net + Reconstruction	82:<3mm 93: 3~10 mm 100: ≥10 mm	0.6	–	0.82 ± 0.15	–
Ottesen et al., 2022 (30)	175/51	–	–	–	Multisequence	HRNetV2 nnUnet	88	1.0 ± 1.1		0.93 ±0.04	N
Huang et al., 2022 (31)	135/32	1503/278	–	–	T1 CE (T1 GRE)	DeepMedic+	93: (α=0.5) 84: (α=0.995)	158: (α=0.5) 1: (α=0.99)	62: (α=0.5) 99: (α=0.99)	0.80: (α=0.5) 0.76: (α=0.99)	–
Ziyaee et al., 2023 (32)	845/206	3482/930	10.5 ± 7.8	–	T1 CE (T1 GRE)	nnUnet	88.4	0.4 ± 1.0	90.1	0.82 ±0.09	–
Ours	103/25	1339	7.3 ± 7.2	0.7 ± 2.1	T1 CE (BB T1WI)	RLK-Unet	81 ± 7:≤10 mm 99 ± 1:>10 mm	1.8 ± 0.2	80 ± 7	0.54 ± 0.08:≤10mm 0.85 ± 0.03:>10 mm	Y

BM, brain metastasis; DL, deep learning; DSC, Dice similarity coefficient; FP, false-positive; T1 CE, contrast-enhanced 3D T1, T1 GRE: T1 gradient-echo.

First, the DL model was based on a BB image. A previous meta-analysis (33) reported the superiority of BB images for the detection of small BMs (<5 mm) because these images suppress the blood signal and have a higher contrast-to-noise ratio, compared to GRE images. In accordance with this finding, RLK-Unet maintained a high sensitivity of 80.84 in detecting small BMs (≤10 mm), whereas previous models showed a relatively lower performance for small BMs (sensitivity: 15–50) (7, 29). Second, we used a few large kernels instead of a stack of small kernels in the CNN. This approach resulted in larger effective receptive field more efficiently, thereby significantly increasing the sensitivity from 84.52 to 88.36 (**Supplementary Material S2**) (23). However, because of trade-off between sensitivity and precision, the precision of RLK-Unet was unfortunately decreased from 80.6 to 68.4. To replenish this, we implemented MHFs, which maximize the contrast between BMs and normal brain tissue, thereby increasing precision. Lastly, the surface mask effectively decreased FPs, by suppressing some blood vessels that were incompletely suppressed in BB images (34). The choroid plexus also frequently mimicked BMs in our model. It was successfully removed using the choroid plexus mask.

RLK-Unet demonstrated a DSC of 0.66 in segmenting BMs. This value is lower than that reported in previous studies (0.77–0.85) (7, 10, 28). We suggest the following explanation for this result: the DSC cannot incorporate the size of the BMs within its score. Only small pixel differences between the GT and the prediction in small BMs may substantially decrease the score (**Figure 3**) (35). In line with this suggestion, our results showed excellent segmentation performance in larger BMs (DSC of large BMs vs. small BMs: 0.85 vs. 0.54). We presume that small pixel differences in the segmentation of small BMs rarely affect the volumetric assessment. The excellent agreement in the volume measurement of the BM between the GT and the prediction in our results also supports our assumption.

Volumetric measurement may provide a more objective and sensitive quantification to evaluate tumor response to treatment than does linear measurement in the current RANO-BM criteria (36). However, it is not clinically feasible because the manual volumetric measurement is a labor-intensive, time-consuming, and complex task (37). The clinical significance of our work lies in the fact that our automated DL algorithm may alleviate these tedious and labor-intensive tasks while maintaining results similar to those of conventional tumor assessment by a radiologist. Cho et al. (38) recently showed the possibility of end-to-end automated treatment response evaluation of BM. However, the sensitivity of BM detection in their system was relatively low (58.0%–80.0%). In addition, their BM segmentation method included internal necrosis, which should be avoided in volumetric measurements. Previous studies have reported that the presence of necrosis in BMs may be an indication of a response to chemotherapy or radiation therapy (14). Furthermore, various imaging characteristics can change during the course of treatment. For instance, patients receiving a combination of tyrosine kinase inhibitors and intracranial radiation therapy are more likely to experience hemorrhages within their BMs (39). Additionally, the values of the apparent diffusion coefficient show alterations before and after chemoradiation therapy (40). As a result, monitoring changes in these imaging characteristics is essential for assessing the treatment effects on BMs. Considering these aspects, our method may offer improved performance and better alignment with real-world clinical scenarios. Based on these perspectives, our method may have better performance and may better reflect real-world clinical settings.

However, RLK-Unet also showed three disagreements with the conventional RANO-BM criteria for treatment assessment (5.1%; 3/58 patients). RLK-Unet may overestimate treatment responses because it records an equivocal enhancement as a true lesion and may underestimate treatment responses because it ignores subtle enhancement after treatment. The incorporation of dynamic information from longitudinal images into the DL algorithm may improve performance. With an in-depth comparison of pre- and posttreatment images, the DL algorithm may better detect subtle changes in tumor size and assess the treatment response more precisely (41).

Our study has some limitations. First, it was a retrospective single-center study, which is insufficient to address variability in scanning techniques and hardware implementation across hospitals. We used five-fold cross-validation for detection and segmentation and a temporally separated internal test set for the treatment response assessment; however, a multicenter study in the near future is required to improve the generalizability of our results. Second, RLK-Unet has some limitations in assessing leptomeningeal seeding, pachymeningeal seeding, and skull metastases because we excluded these factors from our cohort or removed the skull during preprocessing. Third, RLK-Unet was based on patients with lung cancer and may not be applicable to patients with other primary cancers. Finally, in this work, a contrast-enhanced BB T1WI (3D fast spin echo T1-weighted technique) was used for developing our algorithm because a previous study showed that the performance of an algorithm based on 3D BB T1WI was superior to that based on 3D GRE T1WI (sensitivity: 92.6 vs. 76.8) (8). Our study aligns with this result, with sensitivity, DSC, and precision for 3D BB T1WI and 3D GRE T1WI as follows: 86.9, 0.66, 79.6 vs. 53.7, 0.46, 68.7, as shown in **Supplementary Material 7**. Consequently, our algorithm may not be optimally applied to the 3D GRE T1WI sequence, which is more widely used for BM imaging. Lastly, the performance of our algorithm may not be directly compared with previous studies because of a different dataset. However, we ran publicly available algorithms such as 3D U-Net and nnU-Net, which were utilized in prior studies (8, 30, 32), for our dataset, and their performances are inferior to the results of our algorithm (**Supplementary Material S7**). Consequently, we may conclude that RLK-Unet shows a comparative performance for BM detection and segmentation.

## 5 Conclusions

Our developed DL model for the treatment response assessment of BM had more favorable features in clinical practice than did models reported in previous studies. RLK-Unet uses a single modality but shows excellent performance for the detection and segmentation of BMs, even for small metastases. Moreover, our segmentation results very well predicted GT, while avoiding cysts or necrosis, and exactly measured the volumetric tumor burden. The assessment of the treatment response showed good agreement with the decision of the radiologists. We believe that this research takes DL-based BM evaluation to the next level and may facilitate the clinical workflow for radiologists or neuro-oncologists.

## Data availability statement

The original contributions presented in the study are included in the article/**Supplementary Material**. Further inquiries can be directed to the corresponding authors.

## Ethics statement

The studies involving humans were approved by Gangnam severance hospital IRB. The studies were conducted in accordance with the local legislation and institutional requirements. The ethics committee/institutional review board waived the requirement of written informed consent for participation from the participants or the participants’ legal guardians/next of kin because it was a retrospective study. Written informed consent was not obtained from the individual(s) for the publication of any potentially identifiable images or data included in this article because our institutional review board waived the requirement for informed consent because it was a retrospective study.

## Author contributions

SS: Formal Analysis, Writing – original draft. BJ: Data curation, Writing – review & editing. MP: Writing – review & editing. SS: Supervision, Writing – review & editing. HO: Data curation, Methodology, Writing – review & editing. JK: Methodology, Supervision, Writing – review & editing. SL: Project administration, Writing – review & editing. SA: Conceptualization, Funding acquisition, Supervision, Writing – original draft, Writing – review & editing. J-ML: Conceptualization, Funding acquisition, Writing – review & editing.
