# Supplementary material for: Development of RLK-Unet: a clinically favorable deep learning algorithm for brain metastasis detection and treatment response assessment
Source: Front Oncol. 2024 Jan 15;13:1273013. doi: 10.3389/fonc.2023.1273013 (PMC10823345; doi:10.3389/fonc.2023.1273013)
Supplement: Supplementary file 1 [file DataSheet_1.docx]

**Supplemental Material S1**. Flow chart of patient enrollment. BM, brain metastasis; MR, magnetic resonance.


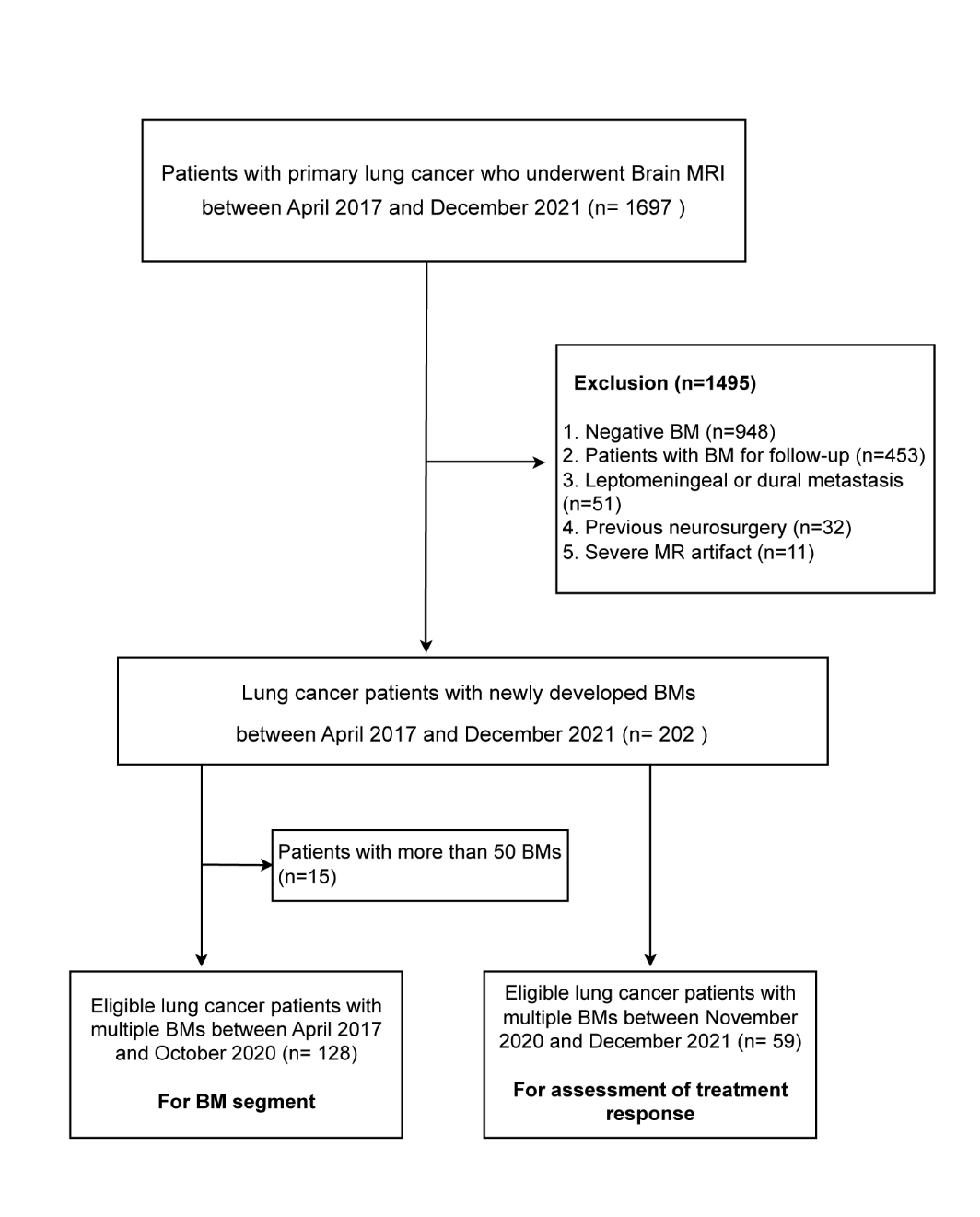


**Supplemental Material S2.** MRI scan parameters for brain metastasis

| Sequence Name | T1-weighted MPRAGE | T2-weighted image | FLAIR | T1-weighted VIBE (CE) | T1-weighted SPACE (CE) |
| --- | --- | --- | --- | --- | --- |
| 2D vs. 3D | 3D | 2D | 2D | 3D | 3D |
| Scan Time | 5min 12sec | 2min 7sec | 2min 42sec | 1min 46sec | 3min 44sec |
| TR(msec) | 2300 | 5300 | 8000 | 9 | 700 |
| TE(msec) | 2.98 | 100 | 128 | 3.69 | 33 |
| TI(msec) | 900 | - | 2370 | - | - |
| Averages | 1 | 1 | 1 | 1 | 1 |
| FOV(mm)  (Read × Phase) | 256 × 248 | 256 × 208 | 230 × 230 | 230 × 230 | 230 × 230 |
| Acquisition Matrix  (Read × Phase) | 256 × 248 | 512 × 324 | 384 × 242 | 224 × 190 | 288 × 288 |
| Flip angle  (E : Excitation)  (R : Refocusing) | 9 (E) | 90 (E)  150 (R) | 90 (E)  150 (R) | 12 (E) | 90 (E)  Variable (R) |
| Parallel Mode  (Phase × Slice) | GRAPPA  2 × 1 | - | GRAPPA  2 × 1 | CAIPIRINHA  1 × 3 | Compressed sensing  9 |
| Pixel Size(mm^2)  (A : Acquisition)  (R : Reconstruction) | 1.0×1.0 (A)  1.0×1.0 (R) | 0.4×0.4 (A) 0.2×0.2 (R) | 0.6×0.6 (A)  0.3×0.3 (R) | 1.0×1.0 (A)  0.5×0.5 (R) | 0.8×0.8 (A)  0.8×0.8 (R) |
| Nr. of Slices | 176 | 24 | 26 | 160 | 240 |
| Thickness(mm) | 1.0 | 5.0 | 5.0 | 1.0 | 0.8 |
| Receive Bandwidth  (Hz/Pixel) | 240 | 222 | 221 | 220 | 423 |
| Echo Train Length | 176 | 19 | 19 | 1 | 30 |
| Echo Spacing(msec) | 7.14 | 11.1 | 9.16 | 9 | 4.68 |

**-** MPRAGE, magnetization-prepared rapid acquisition with gradient echo; FLAIR, fluid attenuated inversion recovery; VIBE, Volumetric interpolated breath-hold examination; SPACE, sampling perfection with application-optimized contrasts by using flip angle evolution

**Supplemental Material S3. Details of the network configuration**

Modified U-Net architecture is a structure that incorporates a large kernel into the encoder and extract feature maps as MHFs from the decoder, as seen in Figure 1. During both training and testing, a 1-channel BB T1WI is inputted. The Input Block is responsible for capturing the details of the 1-channel image, generating 14 feature maps. The RepLK Block of the encoder is composed of a re-parameterized large depth-wise convolution layer and Feed-Forward layer proposed by RepLKNet. (1) Downsampling and upsampling adjust dimensionality using Convolution layer (stride=2) and Transposed Convolution layer, respectively. The Conv Block of the decoder used a small kernel similar to the original U-Net. Subsequently, every decoder layer outputs a 2-channels segmentation map through a convolution layer (1x1x1) and softmax function. Group normalization (2) is implemented after each convolution layer, and nonlinearity is introduced through the use of rectified linear units (ReLU) (3) or gaussian error linear units (GELU) (4).

During training, a loss function is calculated by comparing the segmentation maps outputted from all decoder layers with multi-scale label images, similar to the MHFs approach. (5) The Dice score was used as the loss function. Testing, we evaluated the performance using the Dice Similarity Coefficient (DSC) and sensitivity for individual lesion detection by analyzing only the output segmentation map with the same size as the input image.

In the training process, we used the Adam optimizer (6) with a learning rate of 0.001 and trained the model for a total of 500 epochs. In order to evaluate the performance of the model, we performed training on the dataset by splitting it into 5-folds. In each round of the 5-fold cross-validation procedure, 4 data folds were utilized as training cases, while the remaining fold was utilized for testing. For validation purposes, we randomly selected 10% of the training cases. Particularly, the stratified K-fold method was used to ensure an even distribution of small and large BMs in both the training and test sets. The network implementation was based on PyTorch 1.10.1 and trained on an NVIDIA RTX A6000 GPU with 48 GB of memory. In both training and testing, the input image was cropped to remove the background. Intensity normalization was performed using z-score transformation. Moreover, training data augmentation was applied using the TorchIO (7) Python package.

**Supplemental Material S4.** Bar char showing distribution of size of BMs in our data set. The red dotted line indicates a diameter of threshold of 10 mm between small BM and large BM.

**
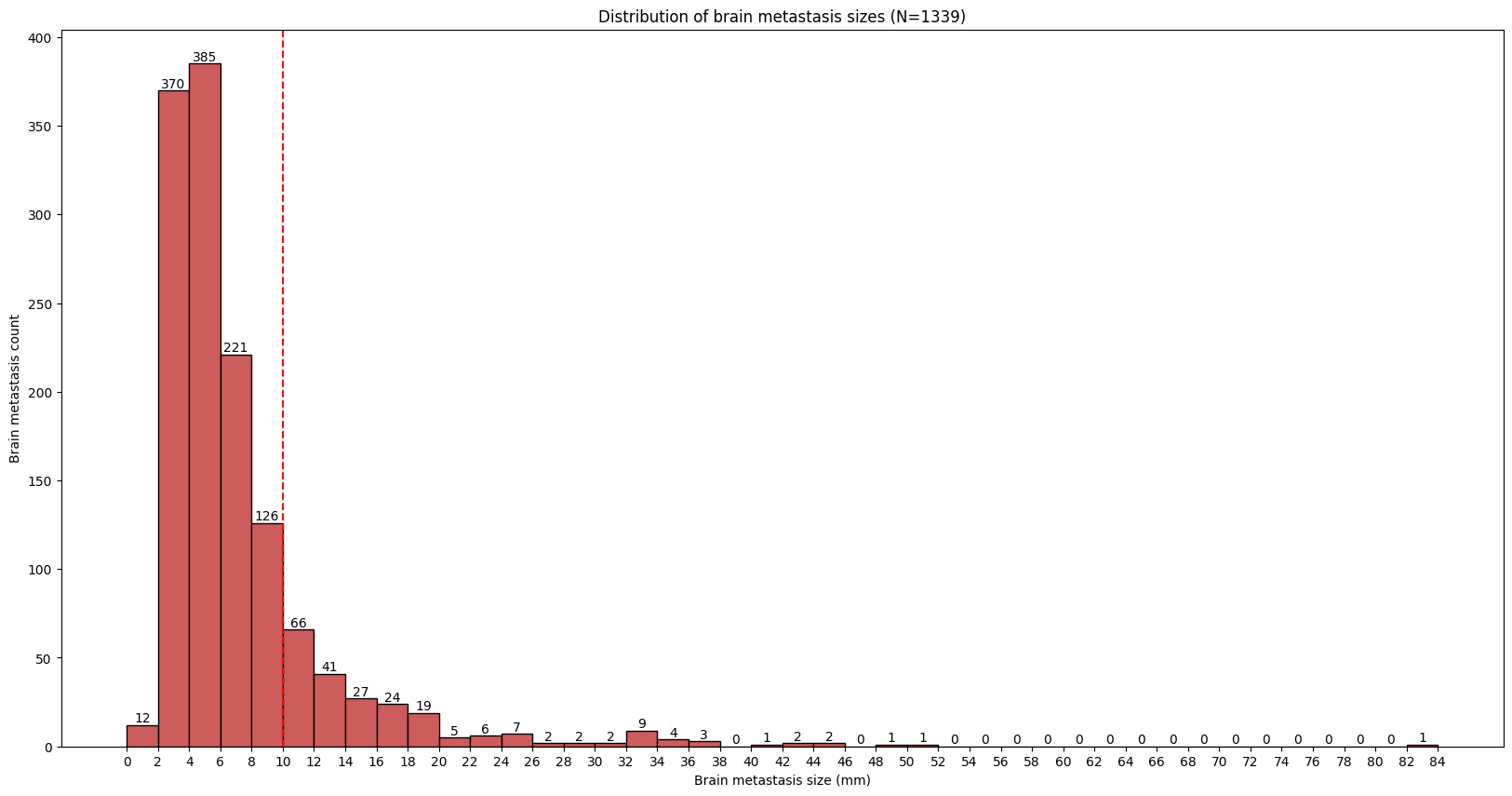
**

**Supplemental Material S5.** BMs size in train and test sets across all folds.


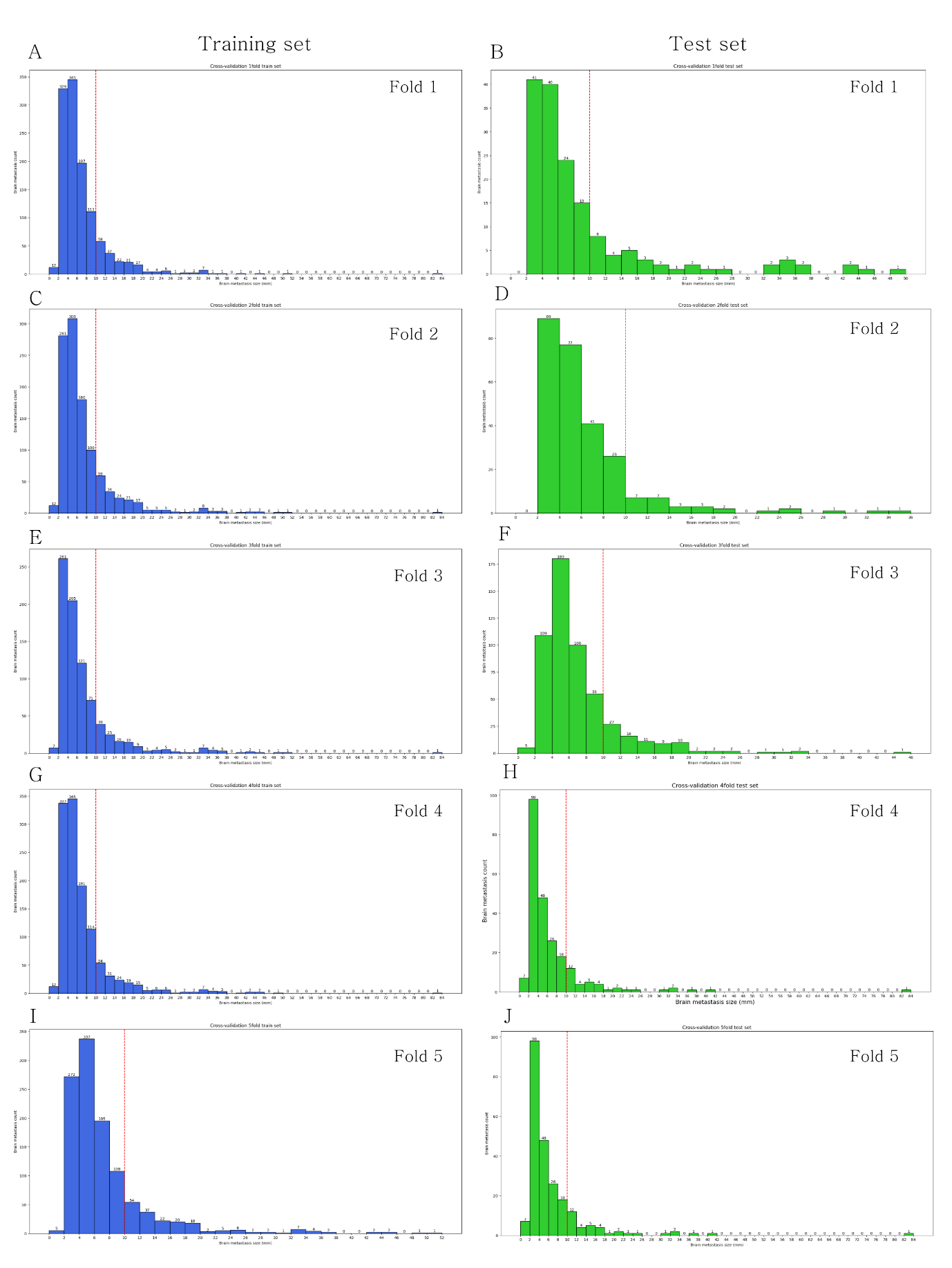


**Supplemental Material S6**. Flow chart of post-processing.


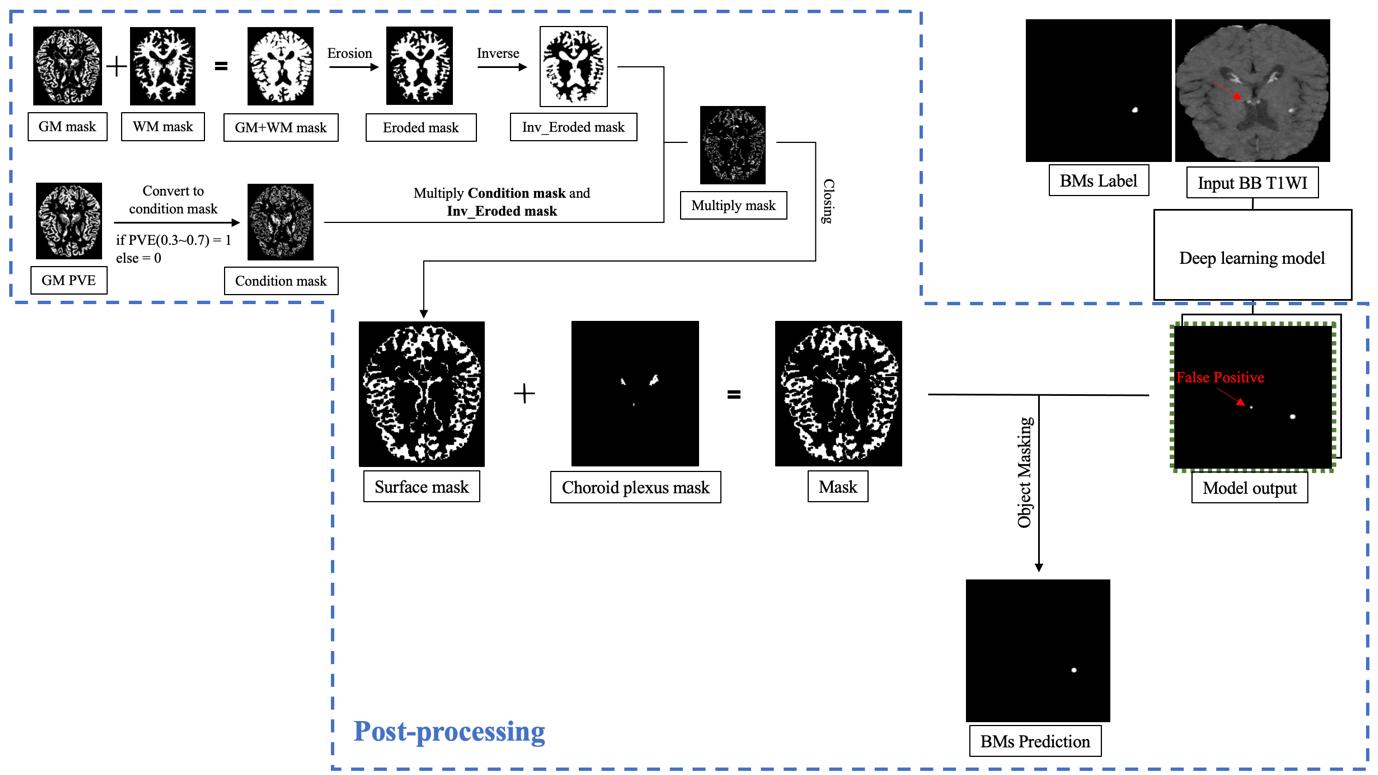


To create the *Surface mask*, a tissue mask is first obtained by combining the GM mask and White Matter (WM) mask. A tissue mask is used to remove the surface where GM and WM interface. Additionally, a *Surface mask* is generated using Partial Volume Effect (PVE) (8), which is commonly observed in medical imaging. PVE is a common phenomenon in medical imaging that occurs due to the limited spatial resolution of the image, which results in a mixture of different tissue types in a single voxel. FMRIB’s Automated Segmentation Tool (Fast) (9) is utilized to obtain the PVE of the GM, where the part to which the GM belongs shows a probability value for each voxel. The probability value of PVE is then used to create a *Surface mask*.

Furthermore, a choroid-plexus segmentation method is employed using Gaussain Mixture Models (GMM) to generate a choroid plexus mask. (10) The final mask image is created by combining the *Surface mask* and *Choroid-plexus mask*. The BMs prediction images are then obtained by masking the final mask image to the foreground channel in the deep learning model output feature. In the masking process, if all voxels of the BMs object predicted in the model output belong to the mask image, they are treated as FPs and removed.

**Supplemental Material S7.** Comparison of performances of deep learning algorithm for brain metastases.

|  | Sensitivity (%) | | | DSC | | | Precision (%) |
| --- | --- | --- | --- | --- | --- | --- | --- |
|  | Small BMs | Large BMs | All | Small BMs | Large BMs | All |  |
| 3D U-Net model^*^ | 79.66 ±9.56 | 96.76 ±3.28 | 84.52 ±7.44 | 0.52 ±0.06 | 0.83 ±0.04 | 0.65 ±0.03 | 80.60 ±8.74 |
| nnU-Net (11) | 78.98 ±4.02 | 95.62±3.98 | 81.68 ±4.69 | 0.55 ±0.08 | 0.85 ±0.07 | 0.64 ±0.04 | 78.98 ±9.80 |
| RepLK block^**^ | 85.54 ±3.42 | 97.38 ±3.71 | 88.36 ±1.96 | 0.54 ±0.04 | 0.85 ±0.04 | 0.66 ±0.02 | 68.40 ±5.36 |
| RepLK + MHFs | 85.16 ±4.23 | 98.9 ±1.13 | 88.40 ±2.78 | 0.54 ±0.08 | 0.85 ±0.03 | 0.66 ±0.02 | 73.96 ±6.29 |
| RLK-Unet (RepLK + MHFs with post-processing) | 80.84 ±7.32 | 98.66 ±1.26 | 86.90 ±4.07 | 0.54 ±0.08 | 0.85 ±0.03 | 0.66 ±0.02 | 79.60 ±6.46 |
| RLK-Unet using conventional contrast enhanced T1WI *** | 41.34±7.44 | 86.04±5.43 | 53.78  ±3.92 | 0.33  ±0.05 | 0.75  ±0.06 | 0.46  ±0.02 | 68.74  ±6.55 |

^*^ 3D U-Net model, where all convolution kernels are 3x3x3.

^**^ U-Net architecture using encoder blocks with a large kernel (13x13x13) applied.

^***^ RLK-Unet using T1-weighted VIBE (CE)

**References**

1. Ding X, Zhang X, Han J, Ding G, editors. Scaling up your kernels to 31x31: Revisiting large kernel design in cnns. *Proceedings of the IEEE/CVF Conference on Computer Vision and Pattern Recognition*; 2022.

2. Wu Y, He K, editors. Group normalization. *Proceedings of the European conference on computer vision (ECCV)*; 2018.

3. Nair V, Hinton GE, editors. Rectified linear units improve restricted boltzmann machines. *Proceedings of the 27th international conference on machine learning (ICML-10)*; 2010.

4. Hendrycks D, Gimpel K. Gaussian error linear units (gelus). *arXiv preprint arXiv:1606.08415* (2016).

5. Park G, Hong J, Duffy BA, Lee J-M, Kim H. White matter hyperintensities segmentation using the ensemble U-Net with multi-scale highlighting foregrounds. *Neuroimage* (2021) 237:118140.

6. Kingma DP, Ba J. Adam: A method for stochastic optimization. *arXiv preprint arXiv:1412.6980* (2014).

7. Pérez-García F, Sparks R, Ourselin S. TorchIO: a Python library for efficient loading, preprocessing, augmentation and patch-based sampling of medical images in deep learning. *Computer Methods and Programs in Biomedicine* (2021) 208:106236.

8. Tohka J, Zijdenbos A, Evans A. Fast and robust parameter estimation for statistical partial volume models in brain MRI. *Neuroimage* (2004) 23(1):84-97.

9. Zhang Y, Brady M, Smith S. Segmentation of brain MR images through a hidden Markov random field model and the expectation-maximization algorithm. *IEEE transactions on medical imaging* (2001) 20(1):45-57.

10. Tadayon E, Moret B, Sprugnoli G, Monti L, Pascual-Leone A, Santarnecchi E, et al. Improving choroid plexus segmentation in the healthy and diseased brain: Relevance for Tau-PET imaging in dementia. *Journal of Alzheimer's Disease* (2020) 74(4):1057-68.

11. Isensee F, Jaeger PF, Kohl SAA, Petersen J, Maier-Hein KH. nnU-Net: a self-configuring method for deep learning-based biomedical image segmentation. *Nat Methods* (2021) 18(2):203-11. doi:10.1038/s41592-020-01008-z
